# Exploiting Waste towards More Sustainable Flame-Retardant Solutions for Polymers: A Review

**DOI:** 10.3390/ma17102266

**Published:** 2024-05-11

**Authors:** De-Xin Ma, Guang-Zhong Yin, Wen Ye, Yan Jiang, Na Wang, De-Yi Wang

**Affiliations:** 1 Liaoning Provincial Key Laboratory for Synthesis and Preparation of Special Functional Materials, Shenyang University of Chemical Technology, Shenyang 110142, China; madexin_0621@163.com (D.-X.M.); na_jiangyan@sina.com (Y.J.); iamwangna@syuct.edu.cn (N.W.); 2 Escuela Politécnica Superior, Universidad Francisco de Vitoria, Ctra. Pozuelo-Majadahonda Km 1.800, Pozuelo de Alarcón, 28223 Madrid, Spain; amos.guangzhong@ufv.es; 3 Sino-Spanish Joint Research Center for Advanced Materials Technology, Shanghai Research Institute of Chemical Industry Co., Ltd., Shanghai 200062, China; yewen12003@163.com; 4 Shanghai Engineering Research Center of Functional FR Materials, Shanghai Research Institute of Chemical Industry Co., Ltd., Shanghai 200062, China; 5 IMDEA Materials Institute, C/Eric Kandel, 2, Getafe, 28906 Madrid, Spain; 6 Shenyang Research Institute of Industrial Technology for Advanced Coating Materials, Shenyang 110142, China

**Keywords:** waste, flame retardant, sustainable, polymeric materials

## Abstract

The development of sustainable flame retardants is gaining momentum due to their enhanced safety attributes and environmental compatibility. One effective strategy is to use waste materials as a primary source of chemical components, which can help mitigate environmental issues associated with traditional flame retardants. This paper reviews recent research in flame retardancy for waste flame retardants, categorizing them based on waste types like industrial, food, and plant waste. The paper focuses on recent advancements in this area, focusing on their impact on the thermal stability, flame retardancy, smoke suppression, and mechanical properties of polymeric materials. The study also provides a summary of functionalization methodologies used and key factors involved in modifying polymer systems. Finally, their major challenges and prospects for the future are identified.

## 1. Introduction

Polymeric materials are essential in daily life, industrial facilities, and medical applications [1]. However, their flammability leads to significant risks to human life and property [2]. As a result, public awareness of the flame-retardant properties of polymers has increased [3,4,5]. The growing awareness of environmental issues, the energy crisis, and advancements in science and technology have led to new requirements for flame-retardant polymeric materials [6,7]. Sustainable flame retardants, obtained from renewable sources and manufactured using environmentally friendly chemical processes, have gained scientific attention [8,9]. These flame retardants have negligible adverse effects on human well-being and the ecosystem, offering a potential solution for enhancing fire safety while maintaining sustainability.

One potential source of flame retardants is waste material. Numerous waste materials include inherent natural chemicals that exhibit flame-retardant properties. By harnessing these materials, it becomes possible to mitigate landfill accumulation while simultaneously establishing a more sustainable reserve of flame retardants. Waste materials that have been studied for their potential use as flame retardants include industry, food and plant waste. Scientists have found that these materials can be effective at slowing down the spread of fire when added to other materials. Researchers have explored the use of agricultural waste materials like rice husk, wheat straw, and maize stalks for their flame-retardant properties. For a typical case, Wang et al. [10] found that adding corn stalk biochar (CSB) to high-density polyethylene (HDPE) could enhance its flame retardancy. Meanwhile, the limiting oxygen index (LOI) remained at 25.5%. The presence of CSB at a 60.0% concentration significantly decreased the peak heat release rate (pHRR) and total heat release (THR) of HDPE composites, with a 46.1% decrease in pHRR and a 44.6% reduction in THR compared to pure HDPE.

**Figure 1:**
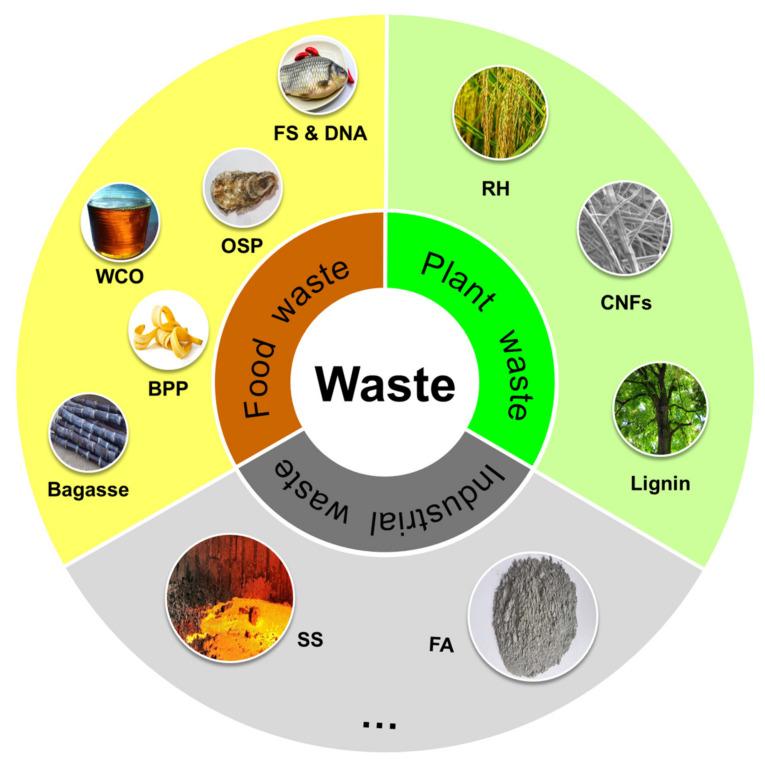
Diagram demonstrating the composition of flame-retardant additives derived from industrial, food, and plant wastes. Fly ash (FA), steel slag (SS), oyster shell powder (OSP), waste cooking oil (WCO), fish scales (FS), banana peel powder (BPP), rice husk (RH), cellulose nanofibers (CNFs).

The increasing generation of waste, including fly ash (FA) [11,12], steel slag (SS) [13], eggshell (ES) [14,15,16], bagasse [17,18,19,20], banana peel powder (BPP), oyster shell powder (OSP) [21], waste cooking oil (WCO) [22], fish scales (FS) [23], coffee grounds [24], rice husk (RH) [25], cellulose nanofibers (CNFs) [26], and lignin, has led to environmental issues. Flame-retardant additives offer potential due to their diverse constituents. Recycling and reusing waste have increased in recent years. As shown in Figure 1, this study focuses on industrial, food, and plant waste and examines their influence on thermal stability, flame retardancy, smoke suppression, and mechanical characteristics of polymeric materials. It also explores the flame-retardant mechanisms of different waste materials, their primary challenges, and potential future opportunities.

## 2. Classification and Application of Waste-Based Flame Retardant

Waste-based flame-retardant additives are sustainable alternatives to traditional flame-retardant additives. They are derived from waste products and can be categorized based on their source or chemical composition. Examples include fly ash, steel slag, eggshell, bagasse, oyster shell powder, waste cooking oil, fish scales, fish deoxyribonucleic acid, coffee grounds, rice husk, cellulose nanofibers, lignin, and so on. These materials are produced from industries, food, or plants.

### 2.1. Industrial Wastes

As civilization advances, industrial waste, including fly ash and steel slag, poses a threat to the environment and human health. However, these wastes have potential for recycling due to their diverse flame-retardant components, making them suitable for manufacturing flame-retardant polymers. Following are some examples.

#### 2.1.1. Fly Ash (FA)

The expansion of the power sector has led to a significant increase in FA emissions, causing significant contamination of land, water, and air [27]. FA is a byproduct of thermal power plants and is primarily composed of silicon dioxide (SiO_2_), aluminum oxide (Al_2_O_3_), and iron oxide (Fe_2_O_3_). The principal uses of FA include building materials, construction, roads, fill material, and agricultural techniques [28,29]. Its unique chemistry and structure have a big impact on its flame retardancy. Scientific experts are interested in the appropriate treatment and exploitation of FA, which is present in various composites (Table 1).

**Figure 2:**
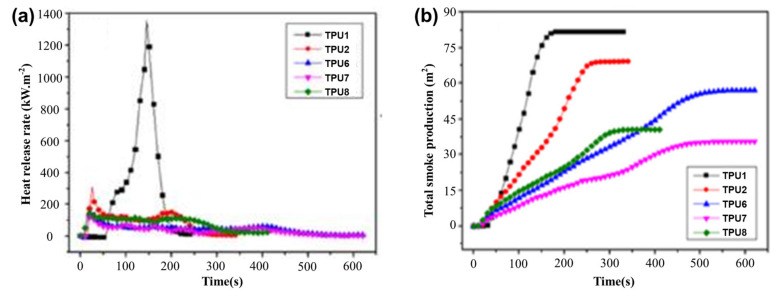
(**a**) HRR and (**b**) TSP curves of pure TPU and TPU composites. Redrawn from [31]. Copyright (2020) Wiley.

**PU.** The addition of FA to polyurethane (PU) was used as a synergistic agent to achieve a specific flame-retardant performance. Usta’s [30] research on rigid polyurethane foams (RPUFs) with FA and an intumescent flame retardant (IFR) showed the total heat release (THR) of RPUF/FA was 19% lower than RPUF. The incorporation of IFR and FA/IFR into RPUF reduced THR by 26% and 33%, respectively. This indicated IFR had a good synergistic effect with FA. Furthermore, Zhou et al.’s [31] study on thermoplastic polyurethane (TPU) showed that substituting IFRs with FA could improve the flame-retardant effect. As shown in Figure 2a,b, the peak heart release rate (pHRR) of TPU/25 wt% IFR composites was reduced by 77.4% compared to pure TPU, and total smoke production (TSP) was reduced by 15.7%. Moreover, the pHRR and TSP of TPU/20 wt% IFR/5 wt% FA were reduced by 91.1% and 56.7%, respectively, compared to pure TPU. Thus, a modest quantity of FA paired with IFRs might improve the fire security of TPU materials.

**EP.** The mixing of FA with epoxy resin (EP) was used as a synergistic agent to obtain a specific flame-retardant effect. Zanoletti et al. [32] found that FA, stabilized through a simple stabilization process, was a promising alternative to traditional flame retardants. Furthermore, the inclusion of FA influenced the electrical attributes of composites. Nguyen [33] examined the additive effects of nanoclay and FA on mechanical properties, flame retardancy, and electrical characteristics. The results showed that nanocomposites had tensile forces of 64.1 MPa, flexural forces of 89.3 MPa, compressive forces of 215.2 MPa, and impact strength of Izod 14.5 kJ/m^2^, with an LOI of 26.8% of fire-retardant material at a combined ratio of 40.0% FA and 3.0% nanoclay. The inclusion of nanoclay in the material produced a winding electric channel, limiting the spread of electric power plants and thereby affecting the electrical properties of the composites.

**Figure 3:**
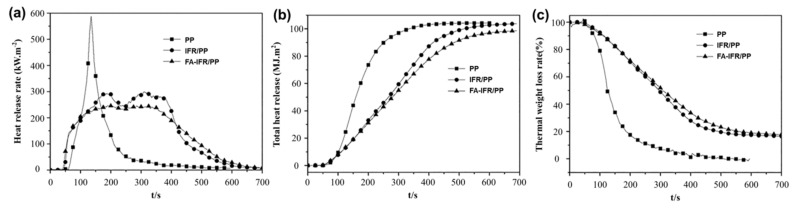
(**a**) HRR, (**b**) THR, and (**c**) thermal weight loss rate curves of PP and flame retardant PP composites. Redrawn from [34]. Copyright (2020) Wiley.

**Polyolefin.** Song et al. [34] used FA as a synergist to improve flame resistance in intumescent flame-retardant PP containing hydroxymethylated lignin. As shown in Figure 3, the addition of 0.5% FA to the polypropylene (PP) composite (0.5FA/IFR/PP) significantly improved flame retardancy, increased the LOI from 28% to 33%, and passed the V-0 rate in the UL-94 test. FA also reduced the pHRR of pure PP by 58% and achieved a higher char residue.

**Figure 4:**
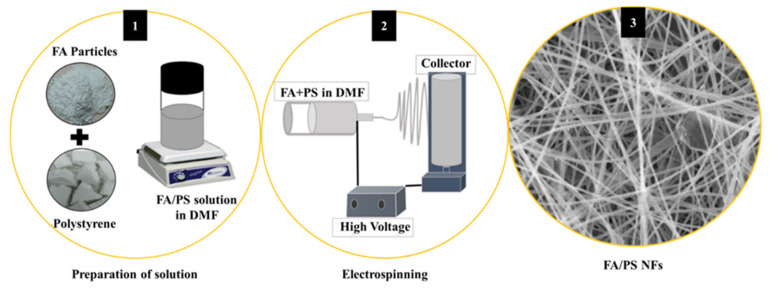
The production of the FA–PS fiber composite is shown graphically: (**1**) preparation of solution, (**2**) electrospinning and (**3**) SEM morphology of the FA/PS nano fibers. Redrawn from [35]. Copyright (2022) MDPI.

**Others.** FA might be present in a diverse range of polymers beyond those previously indicated. (1) Polystyrene (PS). The electrospinning procedure resulted in continuous PS fibers with excellent FA particle dispersion, as shown in Figure 4. Park et al. [35] studied the flame-retardant properties of these composite nanofibers, which increased the LOI value of a polystyrene membrane when FA particles were added. Furthermore, the FA–PS composite membrane shrank and self-extinguished after being removed from the fire source. (2) Ethylene vinyl acetate (EVA). FA might be utilized as a starting material for the synthesis of flame retardants. Li et al. [36] synthesized a smoke-suppression and flame-retardant layered double hydroxide (LDH) containing Mg-Al-Fe ternary, which was investigated in EVA composites. The results showed that most samples had a V-0 rate in the UL-94 test and the highest LOI of up to 28.5%. The EVA sample had the lowest pHRR and THR.

#### 2.1.2. Steel Slag

Steel slag, a common solid waste from industry, is a result of China’s rapid steel industry growth, leading to annual emissions exceeding 100 million tons [37]. Technical constraints hinder its comprehensive utilization, causing it to pollute the environment and encroach on land. Steel slag is primarily composed of metal oxides like silicon oxide (SiO_2_), calcium oxide (CaO), aluminum oxide (Al_2_O_3_), and manganese dioxide (MnO_2_) [38], which can limit the amount of smoke, harmful chemicals, and heat release [39,40,41].

**Figure 5:**
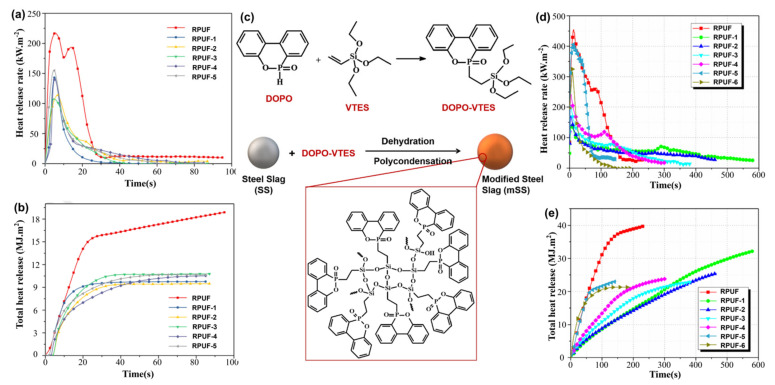
(**a**) HRR and (**b**) THR curves of RPUF samples. Redrawn from [42]. Copyright (2019) Elsevier. (**c**) Synthetic route of microencapsulated steel slag by phosphorus-containing silane modifier (**d**) HRR and (**e**) THR curves of the RPUF samples. Redrawn from [44]. Copyright (2020) Elsevier.

Steel slag was an ingredient in modified rigid PU foam (RPUF) composites (Table 2). Yang et al. [42,43] investigated the utilization of steel slag to modify RPUF with typical flame retardants. As shown in Figure 5a,b, the study found that adding steel slag to RPUF improved thermal stability and reduced heat release, with a 1:1 ratio resulting in lower THR and pHRR compared to the pure sample. As shown in Figure 5c, Tang et al.’s [44] research also showed that when modified steel slag and expandable graphite were mixed into RPUF, the modified steel slag enhanced the rate of expansion while lowering the coefficient of thermal conductivity. Additionally, as shown in Figure 5d,e, a 10% combination of modified steel slag and expandable graphite reduced the pHRR and THR of RPUF composites by 55% and 47%, respectively.

### 2.2. Food Wastes

Food waste, including eggshell, bagasse, banana peel powder, oyster shell powder, waste cooking oil, fish scales, DNA, and coffee grounds, is increasing due to improved living standards. These wastes contain valuable ingredients like calcium carbonate (CaCO_3_), cellulose, hemicellulose, and lignin, which have potential for making flame-resistant polymer compounds and are being explored as potential sources.

#### 2.2.1. Eggshell

Eggshell, an aviculture byproduct, has been found to have reclamation potential due to its chemical composition, low cost, light weight, and environmental advantages [45]. Scientists have applied ES to polymers as synergistic fillers (Table 3), with CaCO_3_ nanopowder being the most common nanofiller in industrial coatings [46,47]. Processed ES may be utilized to replace commercial CaCO_3_ without reducing coating quality [48,49]. Notably, eggshells must be carefully cleaned before being used as fillers. Otherwise, untreated eggshells with various components often alter the flame retardancy of polymers. There are two approaches to including eggshells as synergistic agents: direct addition and adding after conversion.

(1) Direct addition as biofillers

**Figure 6:**
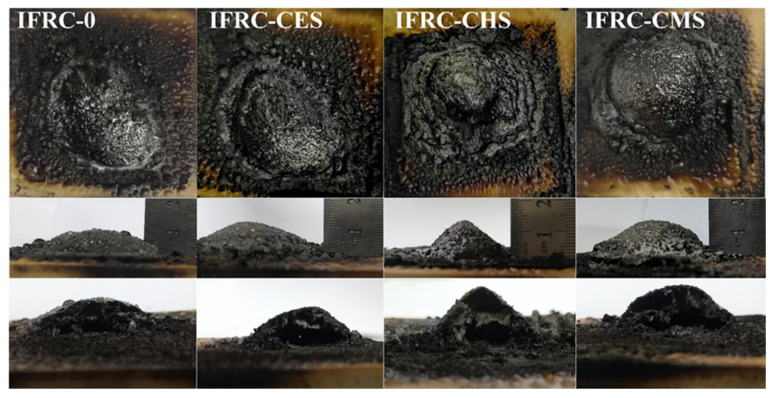
Images of char remain from the huge panel technique test. Redrawn from [53]. Copyright (2021) MDPI.

Eggshell has been employed as a synergistic agent in coatings. Yew et al. [50,51,52] developed an effective intumescent fire-protective coating using eggshell powder as a new biofiller. Water-repellent properties, homogeneous foaming structure, and adhesive strength were all advantages of the coating. A sufficient quantity of nanobiofiller increased fireproofing efficiency as well as mechanical characteristics. Other shell might be utilized in coatings in place of eggshell, which could be employed as a synergistic agent. Wang et al. [53] studied three different shell biofillers: eggshell, conch shell, and clamshell (CMS). They were cleaned, ultrasonicated, and pulverized before being applied to intumescent fire-resistant coatings. The study found that CMS had the highest synergistic impact and decreased pHRR and THR by 23.1% and 32.2%, respectively, as shown in Figure 6.

**Figure 7:**
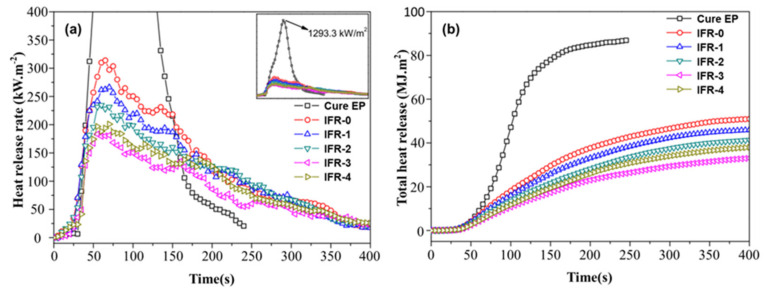
(**a**) HRR and (**b**) THR curves of intumescent fire-proof coatings at 50 kW/m^2^ heat flux. Redrawn from [56]. Copyright (2018) Wiley.

Eggshell can be employed as a fire retardant in polylactic acid (PLA) composites, according to a study by Urtekin et al. [54]. They observed that raising the quantity of eggshell in composites increased its Young’s modulus, thermal stability, and char residue. Furthermore, the LOI was 34.5% with 10% eggshell in composites and was the V-0 level with the eggshell in the IFR system for PLA. The addition of chicken eggshell to intumescent flame retardant (IFR) dramatically decreased heat release and smoke formation, resulting in thermally stable and intumescent char when flaming, according to the research [55]. As demonstrated in Figure 7a,b, the pHRR and THR of EP composites dropped by 42.2% and 35.3%, respectively.

In addition, eggshell might improve the mechanical characteristics of PP composites while also functioning as a fireproof synergist. Younis et al. [57] developed a product using recycled waste polypropylene (WPP) and waste chicken eggshell (WCES) as biofilters. Adding 10 phr of WCES to WPP/WCES composites increased their tensile and flexural strength by 15% and 8%, respectively, compared to WPP composites. Adding magnesium hydroxide ((Mg(OH)_2_) and WCES to the composites increased their tensile and flexural strength, suggesting that WCES and Mg(OH)_2_ collaborate to improve the composite’s mechanical properties.

(2) Adding after conversion to calcium-containing compounds

The production of hydroxyapatite (E-HAP) uses heated ESs to produce calcium oxide (E-CaO). Jirimali et al. [58] discovered that adding E-CaO/E-HAP to linear low-density polyethylene (LLDPE) considerably enhanced the thermal resistance and flame retardancy of the composites. Furthermore, the composite containing E-HAP nanopowder outperformed its excellent mechanical characteristics.

**Figure 8:**
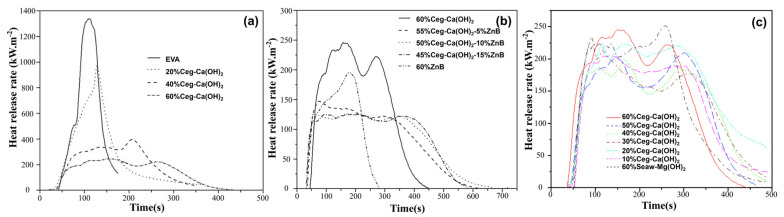
(**a**) HRR curves of EVA containing different content of Ceg-Ca(OH)_2_. (**b**) Evolution of the HRR during cone calorimeter test (50 kW/m^2^) for EVA compositions containing different combinations of Ceg-Ca(OH)_2_ and zinc borate (ZnB). Redrawn from [59]. Copyright (2017) Elsevier. (**c**) HRR curves for EVA/Ceg-Ca(OH)_2_/Seaw-Mg(OH)_2_. Redrawn from [60]. Copyright (2019) Elsevier.

Oualha et al. [59,60] developed a straightforward, quick, and inexpensive technique for converting chicken eggshell waste into lamellar calcium hydroxide particles (Ceg-Ca(OH)_2_). As shown in Figure 8a, the method dropped pHRR by creating dense char. As shown in Figure 8b, the addition of zinc borate reduced the pHRR even more while enhancing the quality of the char. They also created biomaterial calcium hydroxide nanoparticles (Ceg-Ca(OH)_2_) from eggshell collected and magnesium hydroxide nanoparticles (Seaw-Mg(OH)_2_). As shown in Figure 8c, partial replacement of 40 wt% Seaw-Mg(OH)_2_ nanoparticles with Ceg-Ca(OH)_2_ resulted in significant fireproof action and an 85.9% drop in the pHRR of the ethylene–vinyl acetate copolymer (EVA) composite.

**Table 3:** Data of the composites with eggshell as flame-retardant filler.

Polymer	Loading Ratio	LOI (%)	UL-94	pHRR Decrease (%)	THR Decrease (%)	References
EP Coatings	17:1	/	/	23.1	32.2	[53]
PLA	1:1	34.5	V-0	/	/	[54]
EP Coatings	20:1	31.5	V-0	42.2	35.3	[55]
PP	1:1	20.8	HB:12.9 mm/min	/	/	[57]
LLDPE	/	/	HB:20.0 mm/min	/	/	[58]
EVA	1:3	/	/	91.0	/	[59]
EVA	1:2	/	/	85.9	/	[60]

Note: Loading ratio represents flame retardant/eggshell ratio.

In brief, eggshell is high in calcium carbonate and, owing to its inherent qualities, has the ability to function as synergists. However, more research is required to maximize its utilization in various products as well as comprehend its long-term performance.

#### 2.2.2. Bagasse

Bagasse, which is composed of the components lignin, hemicellulose, and cellulose, has potential to enhance mechanical and physical characteristics, prevent combustion, and minimize the usage of synthetic flame retardants [61]. Its academic value lies in its use in coatings and flame-retardancy enhancement for composites, particularly in EP [62,63,64] (Table 4).

**Figure 9:**
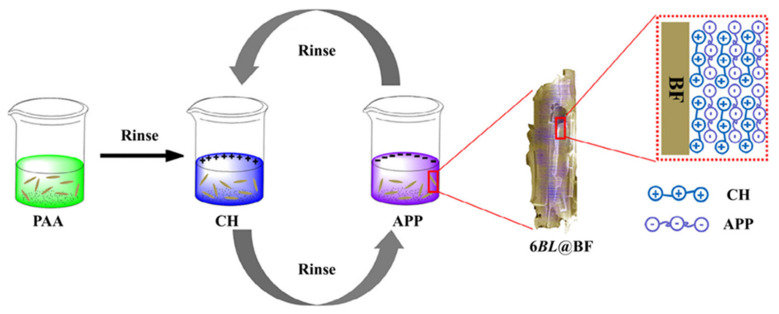
Preparation of chitosan/APP assembled bagasse. Redrawn from [67]. Copyright (2020) Wiley. Notably, PAA is polyacrylic acid.

**EP.** The addition of bagasse to EP was used as a synergistic agent to achieve a specific fire-resistance effect. Shen et al. [65,66] studied the use of agricultural waste bagasse as a synergistic agent to enhance the flame retardancy of EP. They combined bagasse@epoxy of triglycidyl isocyanurate (TGIC)@DOPO with EP to create an interpenetrating network (IPN) composite. The composite was found to be highly flame-retardant. In addition, Chen et al. [67] employed layer-by-layer (LbL) assembly to construct an ecologically friendly fire-resistant EP, demonstrating that the integration of 6BL@BF dropped pHRR and THR by 64.6% and 13.2%, respectively, when compared to unprocessed bagasse (Figure 9). Also, putting chitosan/APP on the surface of the bagasse made it easier for 6BL@BF and the expandable graphite matrix to connect, which made the bend and tensile strength much higher.

**Figure 10:**
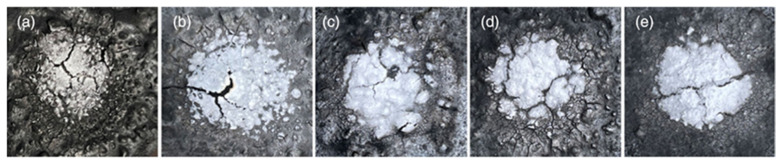
Photographs of char layers during the fire safety test: (**a**) sample SP-0, (**b**) sample SP-1, (**c**) sample SP-2, (**d**) sample SP-3 and (**e**) sample SP-4. Redrawn from [68]. Copyright (2022) Wiley.

**Coatings**. Bagasse, a waste material, can be used in coating systems due to its high flame retardancy. Research by Zhan et al. [68] developed a waterborne intumescent fire-retardant coating using waste bagasse as a filler. As shown in Figure 10, the coating decreased its backside temperature from 397 °C to 223 °C and had a deep char layer with 35.6% carbon content, making it more resistant to oxidation. Furthermore, the coating containing 2% bagasse fared remarkably well in both water-resistant and mechanical characteristics testing.

#### 2.2.3. Banana Peel Powder (BPP)

Powdered banana peel is an agricultural byproduct made from discarded banana peel, which generates 30 million tons of waste annually [69]. There is a plentiful supply of raw materials and it is usable to lessen environmental problems brought on by improper waste disposal [70]. BPP’s main components are cellulose, hemicellulose, and lignin, all with numerous hydroxyls [71]. Its high carbon content makes it suitable for char-forming and flame-retardant additives in PLA and textiles [72] (Table 5).

**Figure 11:**
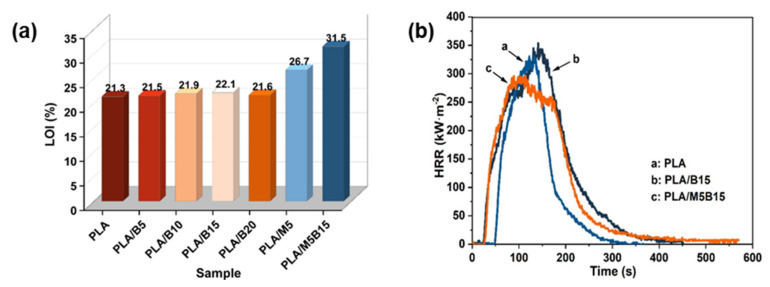
(**a**) LOI results of PLA composites. (**b**) HRR curves of PLA, PLA/B15, and PLA. Redrawn from [73]. Copyright (2022) MDPI.

**PLA.** BPP can be used as a filler in flame-retardant PLA composites, according to a study by Kong et al. [73]. The composites were created using 5 wt% microencapsulated ammonium polyphosphate (MCAPP) and 15 wt% BPP. As shown in Figure 11a,b, the composites demonstrated better thermal resistance, self-extinguishing, and anti-drip properties, and a 10.5% reduction in pHRR. The composites were also helpful in the production of high-quality char in the solid phase and worked as fire retardants in the gas phase. Furthermore, Kong et al. [74] tested a new biobased flame retardant (PA-B) created from BPP and phytic acid (PA). The LOI climbed dramatically to 37.5% when 15.0 wt% PA-B was added to the PLA matrix, achieving the V-0 level in the UL-94 test, and dripping troubles were greatly decreased.

**Textile.** Basak et al. [75] studied BPP, coconut shell extract (CSE), and pomegranate rind extract (PRE) as fire-resistant additives. According to the research, increasing the extract content enhanced the LOI of the treated textile. The burning speed of the PRE-treated textile was 18.29 mm/min, which was much lower than that of the CSE- and BPP-treated textiles. Furthermore, all treated textiles had an attractive natural color and there was no detrimental influence on the tensile strength.

#### 2.2.4. Oyster Shell Powder (OSP)

Oyster shell, a food waste with 96% calcium carbonate, acts as a fire-retardant additive [76]. When decomposed, it produces CaO and CO_2_, which can extinguish fires by blocking oxygen access. Oyster shell powder is popular in composites, particularly when used in conjunction with flame retardants to enhance their flame retardancy.

**Figure 12:**
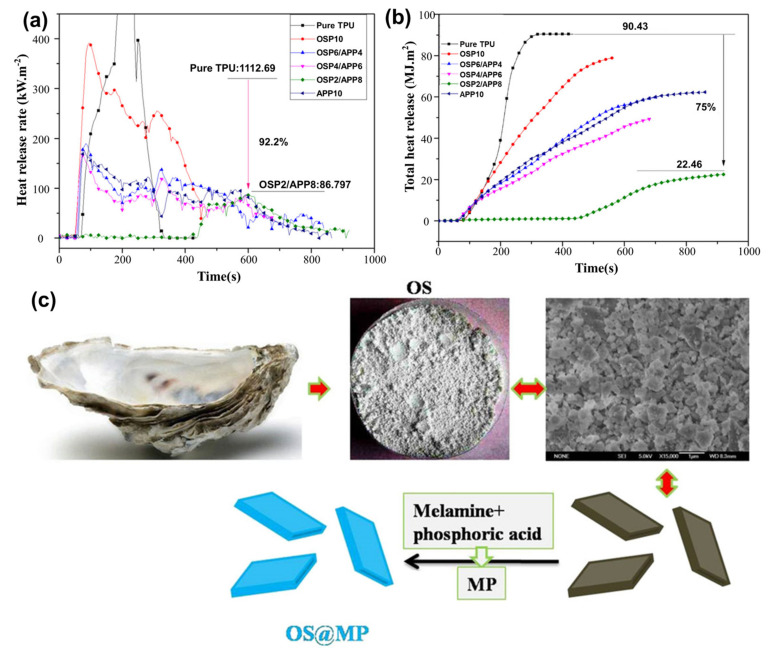
(**a**) HRR and (**b**) THR curve of the composites. Redrawn from [77]. Copyright (2019) Wiley. (**c**) Preparation of OS@MP. Redrawn from [56]. Copyright (2019) Wiley.

The mixing of OSP with TPU was used as a synergistic agent to obtain excellent flame-retardant performance. Chen et al. [56,77,78] studied the synthesis of composites made from OSP and traditional flame retardants like ammonium polyphosphate (APP) and isopropyl titanate. The results showed that OSP and flame retardants effectively reduced smoke and heat release in TPU. A thick carbon layer emerged on the composite surface, preventing flame propagation and minimizing flammable gas production. As shown in Figure 12a,b, (Table 6) the pHRR and THR decreased by 92.2% and 75.0%, respectively. Furthermore, OSP modification also enhanced the flame retardancy of composites. The study also explored OS@MP, a flame-retardant made from OSP and melamine polyphosphate (MP). The noncombustible gases created by OS@MP and the char developed on the composites increased the fire-resistant properties of TPU. The pHRR and THR of the samples with 10.0 wt% OS@MP were reduced by 90.4% and 48.7%, respectively.

#### 2.2.5. Waste Cooking Oil (WCO)

Waste cooking oil is an inexpensive, popular derivative of virgin oils [79], and is produced massively in China, with an average of 500 million tons produced annually [80]. Improper disposal of food waste poses a threat to the environment [81,82], prompting scientific researchers to focus on the efficient treatment and use of cooking oil [83,84,85].

**Figure 13:**
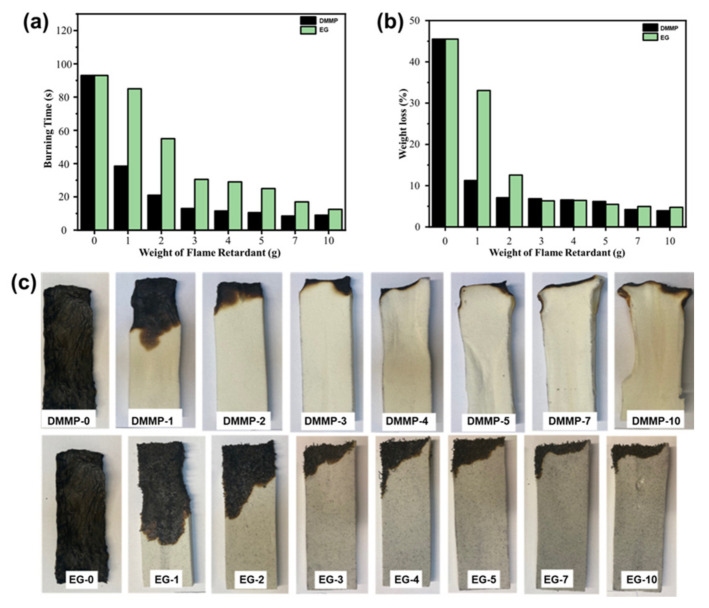
Diagrams depicting the influence of DMMP and EG on the horizontal burning of RPUF in (**a**) burning time and (**b**) reduction in weight after the ignition source was removed. (**c**) Images of WCO–polyurethane foams following horizontal igniting tests: DMMP and EG. Redrawn from [86]. Copyright (2022) American Chemical Society.

Recent research has explored the use of WCO as a potential raw material. Asare et al. [86] created WCO–polyol with a suitable hydroxyl number and the ability to form RPUF. They increased flame retardancy by blending dimethyl methyl phosphonate (DMMP) or expandable graphite (EG) at a higher concentration. The results of the study demonstrated a considerable enhancement in fire resistance, with the WCO-based RPUF igniting in 93 s and losing 46.0% of its weight, as illustrated in Figure 13a,b. As demonstrated in Figure 13c, the addition of 10.7 wt% DMMP decreased igniting time and weight loss to 8.5 s and 3%, respectively, while 16.7 wt% EG lowered igniting time and weight reduction to 12.5 s and 5.0%, respectively. In summary, WCO was processed and used in combination with flame retardants to produce in a flame-retardant composite. As a result, it has massive application potential.

#### 2.2.6. Fish Scales and Fish DNA

Fish scales, which are composed of collagen and hydroxyapatite, are a biological flame retardant owing to their capacity to emit nonflammable gases when burned [87]. These properties reduce the flammability of materials, making them a potential alternative to traditional fire-retardant additives [88]. DNA, a naturally existing and ecologically beneficial fire retardant, is made up of sodium phosphate backbone categories, deoxyribose components, and hydrogen-bonded nucleobases. Researchers have used fish scales and DNA in EP composites to replace hazardous phosphorus or halogen-based additives, making them a promising alternative to traditional additives [89] (Table 7).

FSs can enhance the fire resistance of composites as a synergistic agent. By adding FSs to APP, Liu et al. [90] found that the LOI went from 21.2% to 36.2% and the UL-94 test went from fail to V-0 rate. This indicated that the composite was less likely to catch fire at high temperatures. Furthermore, Zabihi et al. [91,92] employed fishing sector waste DNA to change the structure of clay. A thicker carbon layer might result in a considerable drop in THR and pHRR and a rise in tensile strength. In addition, they modified graphene nanomaterials using DNA waste from the fishing industry. They discovered that adding only 10% of the additives enhanced LOI by 86%, 80%, and 61%, respectively, as well as achieving a V-0 rate in the UL-94 test in EP, PVA, and PS composites. This “multilayer” char residue synergistically enhanced the flame retardancy of polymer nanocomposites.

#### 2.2.7. Coffee Grounds

Coffee grounds, a biodegradable and ecologically benign substance, have been reported to be a rich source of industrially essential sugars and polyphenols [93,94]. The notion of recycling them as polymer fire-resistance fillers is likely to gain attention [95].

Chemical modification of coffee grounds can enhance the flame retardancy of composites. Vahabi et al. [96] developed highly efficient flame-retardant fillers from spent coffee grounds (SCGs) and chemically modified them with phosphorus (P-SCG), resulting in a 39.2% decrease in pHRR and an 11.8% decrease in THR. Furthermore, coffee grounds can also improve the mechanical characteristics and fire resistance of composites. Nguyen et al. [97] studied EP composites containing SCGs, revealing their mechanical characteristics. The inclusion of SCGs enhanced the composite’s tensile strength, flexural strength, impact strength, and compressive strength. In addition, when combined with glass fiber (GF), SCGs might raise the LOI of the composite while simultaneously decreasing the combustion rate of UL-94 HB.

### 2.3. Plant Waste

Plant waste, such as rice husk, cellulose nanofibers, and lignin, is increasing as people’s living standards rise. Because these wastes include important flame-retardant ingredients, studies on their potential usage as fire-resistant fillers in different polymers are being explored.

#### 2.3.1. Rice Husk (RH)

RH is a kind of hull that is used to preserve grains or seeds. It is made of rigid materials, is insoluble in water, and has high silica content. Because of its fireproof components, RH has significant potential in polymers and is employed in a variety of composites (Table 8).

**EP.** RHs with chemical modifications can enhance the flame retardancy of composites. Krishnadevi et al. [98,99] found that functionalizing RHs improved composite flame retardancy. They discovered that amine-terminated cyclophosphazene- and 3-aminopropyltrimethoxysilane-functionalized rice husk ash (RHA) made EP composites better at resisting fire. The special mix of both phosphorous and nitrogen in the phosphazene ring and silica in RHA made the pHRR, THR, and LOI of EP composites much better, and attained a V-0 level in UL-94 test. Meanwhile, the use of RH in the EP composite also provided significant flame retardancy. Kavitha et al. [100] studied the thermal stability and flame-retardant characteristics of an EP composite enhanced with RH. The composite with 11.0 wt% RH showed improved thermal stability and attained a V-0 level in the UL-94 test. Xu et al. [101] studied the use of magnesium phytate (Mg-Phyt) as a biobased flame retardant. They found that combining Mg-Phyt with RHA enhanced its flame retardancy. As a consequence, silica-rich char with excellent thermal stability was produced, decreasing heat release into the EP matrix and flammable gas emissions.

**PP.** Schirp et al. [102] discovered that adding RH to a PP matrix lowered the heat emission rate, resulting in a decline in the pHRR and THR of the composites. Furthermore, Almiron et al. [103] discovered that when volcanic ash and RHA were combined with PP, they boosted the fireproof capabilities of PP, resulting in a decrease in pHRR and THR of PP composites.

**PLA.** Researchers have used chemical modification techniques to study the effect of RH on the flame-retardant properties of PLA composites. Yiga et al. [104] found that modified RHs surpass unmodified RHs in flame-resistant fiber-reinforced PLA composites. Tipachan et al. [105] established a synergy between layered double hydroxide (PKL_DS), rice husk ash silica (SiRHA), and a blend of the two particles that significantly improved PLA’s fireproof capability. PLA nanocomposites with 10 wt% PKL_DS and 5 wt% SiRHA had an LOI of 32.8% and a V-0 level in the UL-94 test with anti-dripping activity.

**Figure 14:**
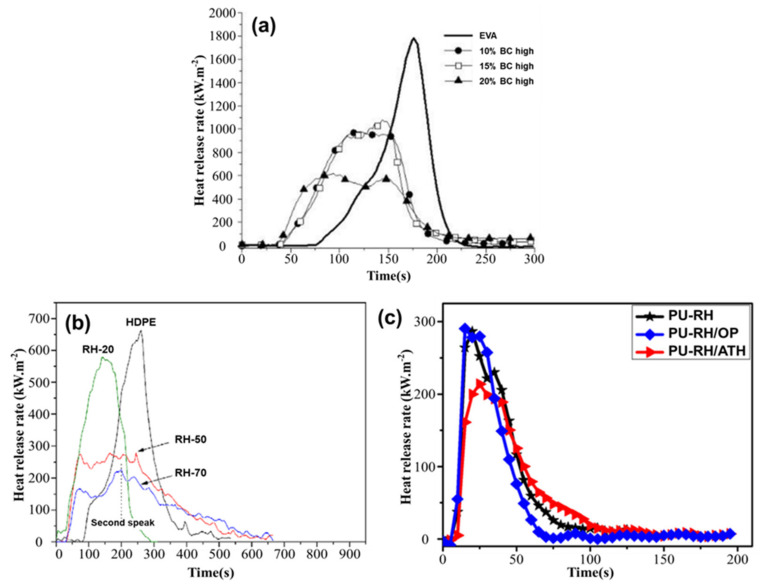
(**a**) HRR curves of EVA and EVA BC high. Redrawn from [106]. Copyright (2021) MDPI. (**b**) HRR curves of HDPE and HDPE–RH composites obtained by CCT at heat flux of 45 kW/m^2^. Redrawn from [107]. Copyright (2009) Elsevier. (**c**) HRR curves of PU–RH, PU–RH/ATH, and PU–RH/OP. Redrawn from [108]. Copyright (2019) MDPI.

**EVA.** Matta et al. [106] investigated three types of biochar: soft wood, oil seed rape, and RH. They mixed biochars at concentrations of 15%, 20%, and 40% in an EVA copolymer. The results showed a decrease in pHRR and THR while increasing additives. The pHRR and THR of EVA composites with 40% RH decreased by 70% and 21%, respectively, as shown in Figure 14a.

**HDPE.** Zhao et al. [107] found that adding RH to polymer composites decreased their flammability. The addition of RH delayed thermal oxidation by 40 °C and provided a flame-retardant effect. HDPE composites with 70% RH exhibited a 65.8% decrease in pHRR and a 22.7% decrease in THR, as shown in Figure 14b.

**PU foam.** The thermal stability, flame retardancy, and mechanical characteristics of RH-reinforced PU foams were examined by Phan et al. [108]. They observed that RHs increased flame retardancy and reduced smoke generation, resulting in a 34.1% decrease in the pHRR of the composite, as shown in Figure 14c.

**Figure 15:**
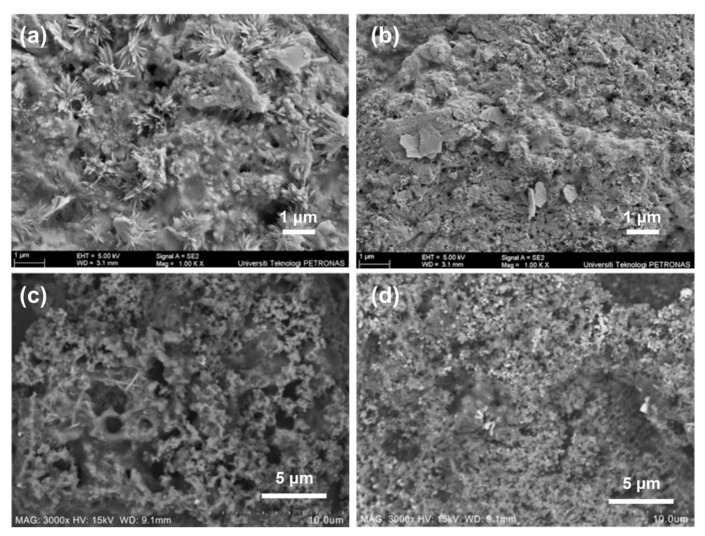
(**a**,**b**) Coating sample before the fire-resistance test and coating sample after the fire-resistance test (**c**,**d**). Redrawn from [111]. Copyright (2021) MDPI.

**Coating.** Nasir et al. [109,110] studied the combustion and thermal stability of an intumescent coating system using rice husk ash (RHS), eggshell, TiO_2_, and Al(OH)_3_. They found that incorporating RHA and TiO_2_ into a waterborne intumescent coating improved fire resistance by reducing HRR and combustion heat. Moreover, Abdullah et al. [111] found that increasing RHA content increased porosity and surface roughness and played a crucial role in the creation of an intumescent char, as shown in Figure 15.

**Table 8:** Data of the composites with rice husk as fillers.

Polymer	Loading Ratio	LOI (%)	UL-94	pHRR Decrease (%)	THR Decrease (%)	References
EP	3:1	58.0	V-0	57.6	31.3	[98]
EP	3:1	62.0	V-0	59.5	64.3	[99]
EP	/	34.0	V-0	/	/	[100]
EP	3:1	22.1	/	28.6	10.6	[101]
PP	7:20	29.3	/	58.9	15.3	[102]
PP	2:1	42.0	/	86.1	61.3	[103]
PLA	2:1	32.8	V-0	/	/	[105]
EVA	/	/	/	70.0	21.0	[106]
HDPE	/	/	/	65.8	22.7	[107]
PU	3:7/7:93	23.0	V-0	34.1	/	[108]

Note: Loading ratio represents flame retardant/rice husk ratio.

#### 2.3.2. Cellulose Nanofibers (CNFs)

Cellulose nanofibers, a sustainable, high-volume fiber of cellulose with sizes ranging from 10 to 100 nm and lengths ranging from a few to tens of micrometers, are gaining interest from researchers and industry due to their abundance, sustainable nature, and excellent mechanical characteristics, which can be used in flame-retardant composites [112,113,114,115,116]. After treatment of the surface, CNFs may be employed for flame-retardant additives in a range of composites (Table 9).

**Figure 16:**
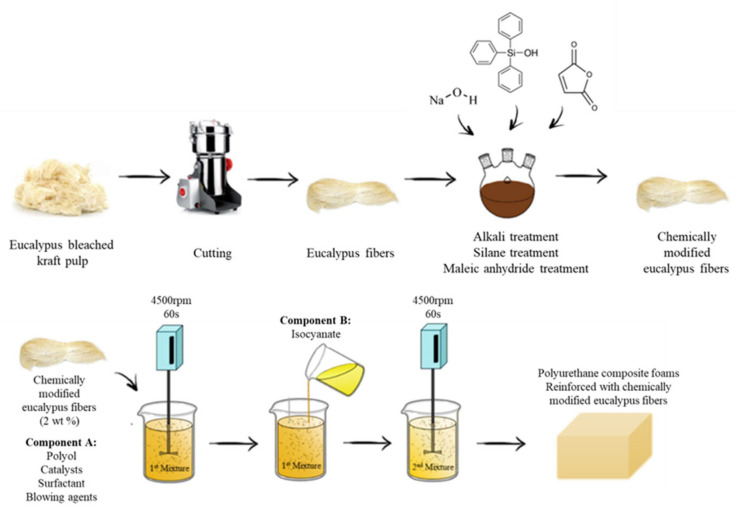
Schematic representation of the preparation of RPUFs modified with eucalyptus fibers. Redrawn from [117]. Copyright (2020) MDPI.

**RPUF.** After surface treatment, CNFs can enhance fire resistance in RPUF composites. Członka et al. [117] discovered that 2% eucalyptus fiber treated with maleic anhydride, alkali, and silane surface modification enhanced the mechanical and thermal characteristics of RPUF, as shown in Figure 16. The silane-treated fibers improved the mechanical characteristics of RPUF composites. Furthermore, the pHRR and TSR of RPUF composites were reduced.

**PLA.** Suparanon et al. [118] found that CNFs can enhance the flame retardancy of PLA composites after surface treatment. They extracted microcrystalline cellulose (MCC) from oil palm empty fruit bunches (OPEFB) and synthesized it as polylactide composite additives. The synergistic effect of tricresyl phosphate (TCP) and OPMC improved the composites’ impact strength and flame retardancy. The composite with the additive had a 38.5% LOI and obtained a V-0 level in the UL-94 test. In addition, Feng et al. [119] studied phosphorus–nitrogen-based polymers on CNFs and came up with PN-FR@CNF, a system that did not catch fire. When 10 wt% PN-FR@CNF was added to PLA composites, they attained a V-0 level in the UL-94 test, their pHRR went down, and their tensile strength improved. The research also discovered that changing CNFs might improve the mechanical characteristics of composites. Furthermore, Yin et al. [120] combined CNFs with green additives to generate APP@CNF, an environmentally friendly fire-retardant additive. The composite, which included 5 wt% APP@CNF, passed the V-0 level in the UL-94 test and had an excellent LOI of 27.5%, which improved flame retardancy. The composite also reduced pHRR and THR by 13.6% and 19.3%, respectively, while increasing the impact strength from 7.63 kJ/m^2^ to 11.8 kJ/m^2^.

#### 2.3.3. Lignin

Lignin, which is plentiful in nature and extensively dispersed in plant-supporting tissues such as wood and bark, has tremendous promise as an ecologically benign flame-retardant resource owing to its excessive carbon content and its multifunctional groups [121,122,123], as shown in Table 10.

**Figure 17:**
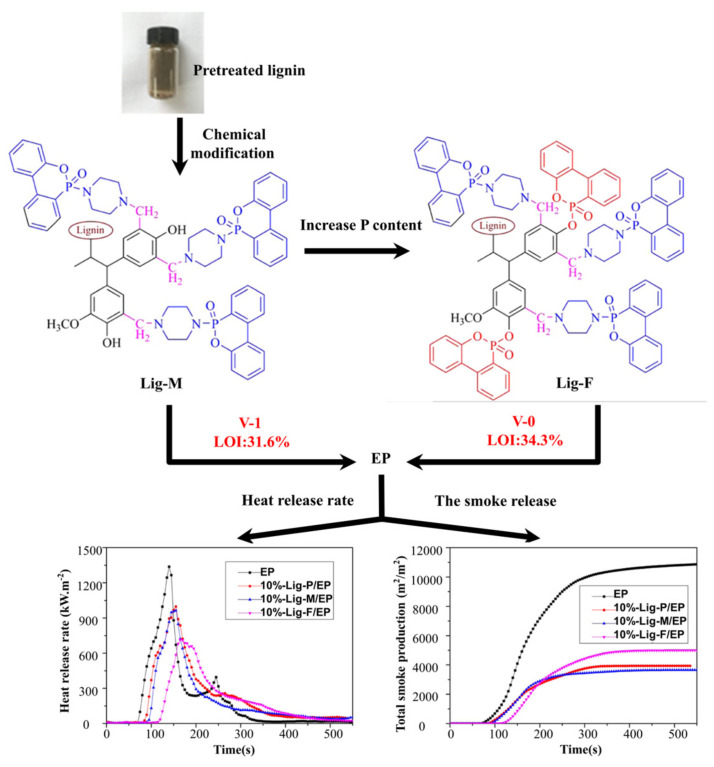
The synthetic route of modified lignin (Lig-M and Lig-F). Redrawn from [125]. Copyright (2020) American Chemical Society.

**EP.** Ding et al. [124] found that straw lignin can be used as a partial replacement for bisphenol A in EPs, resulting in excellent thermal stability. In addition, Dai et al. [125] reviewed lignin with high smoke-suppression capabilities utilizing modified biomass. A Lig-F/EP composite with high phosphorus content achieved the best flame retardancy, obtaining a V-0 level in the UL-94 test and reducing pHRR and the generation of smoke by 46.6% and 52.8%, respectively, as shown in Figure 17.

**PLA.** The flame retardancy of lignin might be enhanced by grafting modification. Yang et al. [126] produced lignin-derived multifunctional bioadditives (TP-g-lignin) by grafting a phosphorus/nitrogen-containing vinyl monomer (TP) to a lignin. The addition of 5 wt% TP-g-lignin to PLA achieved a V-0 level in the UL-94 test. Furthermore, Liu et al. [127] investigated a lignin-derived flame retardant by grafting polyphosphoramide onto lignin. The composite with 8 wt% lignin-derived additives obtained an LOI of 25.8% and a V-0 level in the UL-94 test and reduced THR by 8.4%.

**PP.** Liu et al. [128] investigated a biobased flame retardant derived from conventional lignin grafted with P, N, and copper components for wood–plastic composites. They discovered that functionalized lignin (F-lignin) was more efficient than unmodified lignin (O-lignin) in enhancing thermal stability and flame retardancy. F-lignin slowed combustion, decreased heat release, and lowered smoke generation. Composites containing 5 wt% F-lignin PP reduced pHRR by 9% and THR by 25%, respectively.

**PA.** In their study of the flame retardancy of polyamides (PAs) using kraft lignin and the APP synergistic effect, Cayla et al. [129] discovered that kraft lignin slowed the thermal decomposition of PA composites and lowered pHRR by 66.0% compared with pure PA.

**Table 10:** Data of the composites with lignin as fillers.

Polymer	Loading Ratio	LOI (%)	UL-94	pHRR Decrease (%)	THR Decrease (%)	References
EP	/	34.3	V-0	46.6	8.1	[125]
PP	1:5	/	/	9.0 ± 0.7	25.0 ± 0.8	[128]
PA	/	/	V-2	66.0 ± 0.1	13.0 ± 1.0	[129]

Note: Loading ratio represents flame retardant/lignin ratio.

### 2.4. Other Wastes

In addition to the three types of waste mentioned above, many other wastes contain valuable flame-retardant components. As a result, they could be applied in the field of polymer flame retardants [130]. The flame retardancy of composites is somewhat impacted by wool and biochar compositions. Das et al. [131] investigated biochar and wool composites in conjunction with a halogen-free flame retardant. The results showed that biochar and wool composites significantly lowered the pHRR, produced less smoke, and had a higher mass loss rate than pure PP. Furthermore, wool hybridization improved LOI. The pHRR and THR of the composites decreased by 73.3% and 9.0%, respectively. In addition, certain biobased waste has shown positive outcomes in conventional intumescent flame-retardant coatings. Wang et al. [132] investigated conch shell biofiller (CSBF), which was created by washing, ultrasonically pulverizing, and pulverizing conch shell, and then used in waterborne intumescent flame-retardant coatings. The pHRR and THR decreased by 24.8% and 29.6%, respectively, when compared to a reference sample. As a consequence, CSBF increased the coatings’ heat stability and formation of char performance. Furthermore, the flame retardancy of the composite was also increased by adding waste foam that was intrinsically flame-resistant. Wang et al. [133] investigated thermoset polymer foam waste leftovers by pulverizing melamine formaldehyde (MF) foam with intrinsic fire resistance and adding it as the flame-retardant filler to PUF. The researchers discovered that introducing MF foam powder might greatly lower the HRR and combustibility of PU foam without sacrificing mechanical qualities. Natural fibers derived from renewable resources have relatively low manufacturing costs and are completely biodegradable, providing great benefits for the final qualities of the composites [134,135]. Sanchez-Olivares et al. [136,137,138] researched natural keratin fibers, coconut fibers and agave fibers for fillers in thermoplastic starch–polyester. The results showed that the composites had a good flame-retardant effect. Leather is among the most ancient, widely used materials worldwide. Moreover, feathers contain abundant keratin fibers. Furthermore, feathers are full of keratin fibers. Wrześniewska-Tosik et al. [139,140] studied combinations of elastic polyurethane (EPUR) with milled chicken feathers. The findings showed that composites containing feathers might increase their flame retardancy. Additionally, Battig et al. [141] investigated the use of leather waste (LW) as filler in flame-retardant composites composed of polymers. The findings indicated that EVA composites incorporating LW had 53.0% lower pHRR than pure EVA.

## 3. Conclusions and Perspective

In summary, waste-based flame retardants have seen rapid development in recent years. Due to the different flame-retardant components in certain industrial wastes, food wastes, and plant wastes, they have been employed in the development of flame-retardant polymeric materials. These studies suggest that wastes could be promising alternative flame-retardant materials, particularly for use as flame-retardant additives or synergists, and show the following main advantages and significance.

(i) Reducing waste: By using waste materials as the source of additives for flame-retardant actions, we can reduce the amount of waste that goes to landfills or incinerators. This is important because waste disposal may cause serious environmental problems.
(ii) Cost-effective: Using waste materials to make composite flame retardants is an economical and promising method, since raw resources are often less costly than virgin materials. The utilization of waste materials to make sustainable flame-retardant compounds would aid in waste reduction and the promotion of a circular economy.
(iii) Sustainable: Using waste materials to make flame-retardant substances is a sustainable technique that would help to lessen our dependency on nonrenewable resources.

Researchers limit the quantity of waste that goes to dumpsters while simultaneously developing more sustainable and ecologically friendly products by using the aforementioned waste elements to generate useful flame retardants. However, the research on utilizing waste materials as flame retardants is still in its early stage. There are currently few mainstream waste flame retardants that achieve high flame-retardant efficiency when used alone. In addition, the current limitation on such flame retardants is that the introduction of these fillers often does not bring high value-added functions to the composites, such as improvement in mechanical performance. To further develop novel high-performance waste-based flame retardants, we proposed the following.

(1) Developing possibilities for more types of waste utilization. More waste-based flame retardants should be produced and investigated. Agricultural wastes such as corn stalks can be converted into biochar, which has high flame-retardant properties, while other natural materials, e.g., cellulose-based wastes, which can be extracted from corn cobs and wheat straw, and lignocellulosic wastes, such as sawdust and wood chips, can be used to produce sustainable flame retardants.
(2) Comprehensive analysis of the performance and efficacy of waste derivative flame retardants. In order to screen or develop waste-derived flame retardants with high performance, versatility, and significant economic value, researchers need to conduct a comprehensive cost-effectiveness analysis of objective flame retardants, which can include a systematic evaluation of mechanical properties, cost analysis, flame retardancy, and other possible value-added functions. It is worth pointing out that life cycle assessment (LCA) can be used as a practical and systematic method for evaluating related flame retardants. LCA is a well-known process that is documented in international guidelines (ISO 14040, ISO 14044). The socioeconomic and environmental consequences of the whole value chain for any kind of waste-based flameproof product should be examined using LCA and recognized criteria.

Overall, sustainable flame retardants will play a critical role in achieving a balance between fire safety, cost, and environmental concerns. Ongoing research and development efforts in this area will be key to finding safe, effective, and sustainable solutions for waste flame-retardant additives.

## Figures and Tables

**Table 1:** Data of the composites with fly ash as fillers.

Polymer	Loading Ratio	LOI (%)	UL-94	pHRR Decrease (%)	THR Decrease (%)	References
RPUF	1:1	/	/	19.5 ± 0.8	33.0 ± 0.8	[30]
TPU	3:2	/	/	84.8	55.5	[31]
EP	3:20	/	V-2	/	/	[32]
EP	3:40	26.8 ± 0.3	HB:14.01 mm/min	/	/	[33]
PP	59:1	33.0	V-0	58.0	5.2	[34]
PS	/	24.0	/	/	/	[35]
EVA	/	28.5 ± 0.1	V-0	89.6 ± 0.6	24.0 ± 0.7	[36]

Note: Loading ratio represents flame retardant/fly ash ratio.

**Table 2:** Data of the composites with steel slag as fillers.

Polymer	Loading Ratio	LOI (%)	UL-94	THR Decrease (%)	References
RPUF	1:1	22.0 ± 0.5	/	44.4 ± 0.4	[42]
RPUF	1:1	23.2	V-0	29.7	[43]
RPUF	1:1	24.0	V-0	47.0	[44]

Note: Loading ratio represents flame retardant/steel slag ratio.

**Table 4:** Data of the composites with bagasse as fillers.

Polymer	Loading Ratio	LOI (%)	UL-94	pHRR Decrease (%)	THR Decrease (%)	References
EP	/	29.0	V-0	/	/	[65]
EP	/	29.0	V-0	/	/	[66]
EP	3:7	24.1 ± 0.2	V-1	64.6	13.2	[67]

Note: Loading ratio represents flame retardant/bagasse filler ratio.

**Table 5:** Data of the composites with banana peel powder as fillers.

Polymer	Loading Ratio	LOI (%)	UL-94	pHRR Decrease (%)	THR Decrease (%)	References
PLA	1:3	31.5	V-0	10.5	/	[73]
PLA	/	37.5	V-0	/	/	[74]
Textile	/	32.0	HB: 18.29 mm/min	/	/	[75]

Note: Loading ratio represents flame retardant/BPP ratio.

**Table 6:** Data of the composites with oyster shell powder as fillers.

Polymer	Loading Ratio	LOI (%)	UL-94	pHRR Decrease (%)	THR Decrease (%)	References
TPU	4:1	30.0	V-0	92.2	75.0	[77]
TPU	/	23.0	/	67.5	13.0	[78]
TPU	/	/	/	90.4	48.7	[56]

Note: Loading ratio represents flame retardant/oyster shell powder ratio.

**Table 7:** Data of the composites with fish scales and DNA as fillers.

Polymer	Loading Ratio	LOI (%)	UL-94	pHRR Decrease (%)	THR Decrease (%)	References
EP	2:1	36.2 ± 0.2	V-0	/	/	[90]
EP	/	/	/	20.9	31.2	[91]
EP	/	39.0	V-0	/	/	[92]
PVA	/	32.8	V-0	/	/	[92]
PS	/	28.4	V-0	/	/	[92]

Note: Loading ratio represents flame retardant/fish scales or DNA ratio.

**Table 9:** Data of the composites with cellulose nanofibers as fillers.

Polymer	Loading Ratio	LOI (%)	UL-94	pHRR Decrease (%)	THR Decrease (%)	References
RPUF	/	22.1	/	55.1	8.1	[117]
PLA	2:1	38.5	V-0	/	/	[118]
PLA	/	/	V-0	31.4 ± 0.7	7.7 ± 1.0	[119]
PLA	/	27.5	V-0	13.6 ± 0.6	19.3 ± 1.0	[120]

Note: Loading ratio represents flame retardant/flame retardant additive ratio.

## Data Availability

Data available upon reasonable request.
